# The Role of Attention in a Joint-Action Effect

**DOI:** 10.1371/journal.pone.0091336

**Published:** 2014-03-18

**Authors:** Silviya P. Doneva, Geoff G. Cole

**Affiliations:** Department of Psychology, University of Essex, United Kingdom; University of California, Merced, United States of America

## Abstract

The most common explanation for joint-action effects has been the action co-representation account in which observation of another's action is represented within one's own action system. However, recent evidence has shown that the most prominent of these joint-action effects (i.e., the Social Simon effect), can occur when no co-actor is present. In the current work we examined whether another joint-action phenomenon (a movement congruency effect) can be induced when a participant performs their part of the task with a different effector to that of their co-actor and when a co-actor's action is replaced by an attention-capturing luminance signal. Contrary to what is predicted by the action co-representation account, results show that the basic movement congruency effect occurred in both situations. These findings challenge the action co-representation account of this particular effect and suggest instead that it is driven by bottom-up mechanisms.

## Introduction

Joint-action processes have generated a considerable amount of interest amongst cognitive psychologists over the past decade or so. This work has often shown that acting together with another individual on a task differs from individual performance on the same task [1]–[4]. Furthermore, joint-action phenomena reflect many everyday situations where coordination and synchronization between individuals is often required.

One of the most popular paradigms to study joint-action generates the so-called Social Simon effect (also known as the interactive/joint Simon task), first reported by Sebanz and colleagues [3]. The task is carried out jointly by two individuals with one of them responding to the appearance of, say, a particular colour by pressing a left key, whereas the other presses a right key when a different colour is displayed. Typical results reveal a basic “Simon effect”; participants are quicker to respond to stimuli appearing on the side of the display associated with their button (e.g., left key press, a stimulus appearing to the left). The standard Simon task, in which one participant makes both left and right responses, is usually explained by the event coding approach [5]. According to it, perception and action share a common representational system and actions are therefore coded by their perceivable effects. Thus, response facilitation arises when the stimulus is compatible with the action. Conversely, a stimulus-response mismatch creates competition between the stimulus-primed location and the location that requires a response [6]. Consequently, the Simon effect is only present when two participants share the task (social version) or when an individual performs alone but operates both responses (standard version). However, the effect is abolished in the single-participant Simon paradigm where the participant operates only one of the buttons [7].

A considerable amount of research has examined the properties of the Social Simon effect since for some time it has been considered a signature joint-action phenomenon. The action co-representation account, put forward by Sebanz et al. [3], [4] is an appealing explanation for the observed slowing down of responses, following a stimulus-response mismatch. According to this theory individuals represent their partner's actions irrespective of their own target and even in situations when ignoring the partner's task would have been more beneficial [1]. In terms of brain structures, the human parietal and premotor regions are believed to comprise the action observation network, also known as the mirror neuron system (MNS) [8], [9]. However, more recent research has suggested that it could extend to other cerebral parts as well [10], [11]. For example, in an fMRI study, which investigated the neural basis of perceptual bias on action, a network of five regions was found to subserve the effect [11]. Although most activation occurred in the mirror neuron network, activation was found in other areas, such as the primary motor cortex and the inferior frontal gyrus. The authors also suggested that since implicit perception and explicit action observation/imitation activated the same cerebral network, it is plausible to conclude that this network automatically responds to action observation. Moreover, action mirroring has become a popular mediating mechanism explanation for other joint-action effects [12]. However, although the MNS is believed to be the predominant neural correlate of joint-action, some researchers have expressed doubts not only about its role in action understanding but also about its existence per se [13], [14].

Recently, evidence has been reported which challenges the idea that the Social Simon effect is “social” in nature. Indeed, it is difficult to reconcile how an effect, believed to depend on automatic action co-representation still occurs when no online visual or auditory feedback about the partner has been made available [15]. Furthermore, Dolk and colleagues [16], [17] showed that no partner is required for a Social Simon-like effect to occur. In a modified version of the task, involving the rubber hand illusion [18], Dolk et al. [16] demonstrated that the effect increased when there was a greater difference between the actions of the two co-actors. However, the opposite would be expected if automatic action co-representation was driving the phenomenon, as suggested by Sebanz and colleagues [3], [4]. In addition, the Social Simon effect was found even when the partner was not actively involved in the task and most importantly – when there was no partner at all, only the stroking device, used for the rubber hand illusion, was in operation. In a follow-up paper, Dolk and collaborators [17] again demonstrated that social actors were not necessary for the effect to occur. In a series of experiments, the effect was still observed when different attention-capturing events replaced the co-actor. For instance, in one experiment participants performed the task alongside objects which possessed no biological features, such as a clock and a metronome. This follows previous joint-action work in which the biological partner is either replaced by a non-biological imitation of a real partner (e.g., a wooden hand) [19], or a computer [20]. The Dolk et al. (2013) findings were explained with the referential coding theory [21], according to which stimuli are spatially coded in reference to other events that are either voluntarily attended to or salient enough to attract attention. Thus, the alternative response location in the Social Simon condition is thought to be coded in reference to the person, object or event that occurs there.

This line of research leaves open the possibility that other joint-action effects might be due to bottom-up processes, rather than action co-representation. To assess this we conducted two experiments using another joint-action paradigm commonly employed [22]–[30]. In the basic procedure, two participants sit opposite each other across a table (that incorporates a flat touch screen monitor) and take turns to reach out and touch one of two targets that appear on either the left or right hand side of the workspace. Typical results show that reaction time (RT) is shorter when a participant's target position requires them to make the same reaching action as the one their co-actor just performed. Thus, for instance, if Participant A reaches to their right (because their target appeared there) Participant B will be quicker to reach to their own right. Most authors propose that action co-representation mechanisms contribute to the effect, at least in part, with some suggesting that the effect is solely due to processes that give rise to action congruency effects [24]. Such effects are known to occur and have been demonstrated with a variety of actions [31], [32]. Thus, when Participant A reaches to their right, Participant B is said to be quicker to reach to their own right because this is a congruent mirroring action within an egocentric framework.

In the present Experiment 1 we examined whether this particular movement congruency effect would still occur even if the co-actors used different parts of their body to make a response, and thus no action congruency or mirroring could take place. Experiment 2 then examined the “socialness” of the basic effect by assessing whether another person was even necessary to induce the phenomenon.

## Experiment 1: Acting with a Co-actor, Responding with Their Arm or Foot

Recall that the action co-representation account proposes that co-actors in joint-action tasks “form shared representations of tasks quasi automatically” and that “the other's task … [is] … represented in a functionally equivalent way to one's own” [33], p.72. Furthermore, this is thought to be subserved by the MNS which has been found to be active both during action execution and action observation in humans and monkeys [34]. It follows therefore that if the two co-actors use different parts of their body to respond, no movement congruency effect should be observed because different actions are being performed. In other words, a movement congruency effect should not occur if the observed and the required actions mismatch not only visually but also kinesthetically. Indeed, research on action co-representation suggests that some actions are only simulated when the two co-actors are similar enough [35], [36]. In Experiment 1 we employed a variant of the standard arm movement congruency effect described in the Introduction in which participants reached with their hand/arm to the target location. Importantly, their (confederate) co-actor either also used her hand/arm to respond or her leg/foot.

### Methods

#### Ethics Statement

Ethical approval from the ethics committee of the University of Essex was obtained prior to commencement of the two experiments. All participants gave their written informed consent to take part in this research.

#### Participants

A volunteer sample of 21 (9 male; 12 female) participants aged between 20 and 45 (*M* = 25.38 years, *SD* = 7.05 years) took part. All of them were students at the University of Essex who participated in exchange for £4. All were right-handed and were naïve to the purposes of the study.

### Stimuli and Apparatus

**Figure 1:**
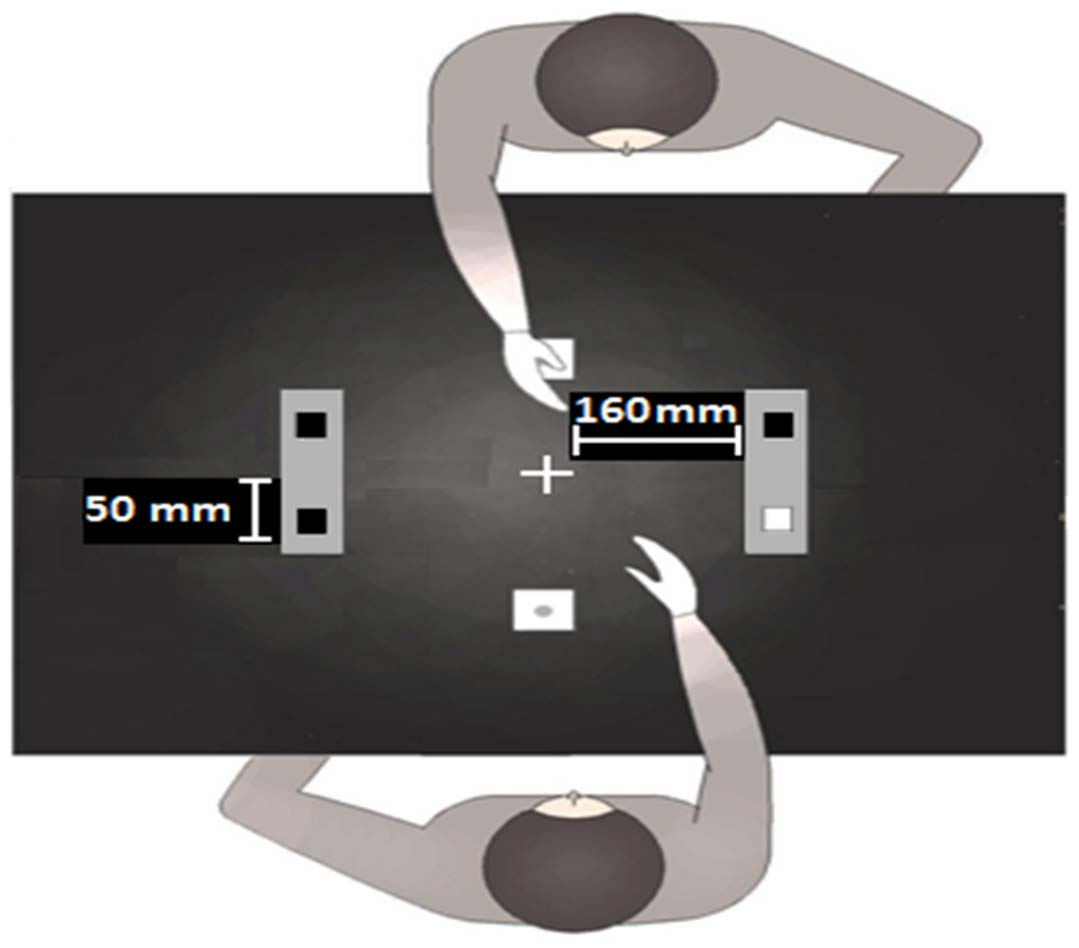
An illustration of the standard condition in the movement congruency paradigm used in Experiments 1 and 2. Each person takes turns to reach out and touch one of two targets presented on the left or right. In the figure shown, one person is reaching to their right where the target has illuminated.

The stimuli were displayed on a 19.5-inch LCD touch-screen monitor built flat into a table, raised 74 cm from the floor. They were presented against a uniform white background (74.6 cd/m^2^). The two co-actors sat facing one another such that the distance between their chests and their “home buttons” was approximately 160 mm (See Fig. 1). In the foot condition, the confederate sat on a chair raised 58 cm from the floor, whereas participants in all conditions and the confederate in the hand condition were seated at a height of approximately 44 cm. The 4 stimulus locations were denoted by 4 black squares (19.6 mm^2^ each) which acted as “placeholders”, and remained present for the entire trial duration. Two placeholders (1 to the left, 1 to the right), located at a distance of 160 mm from the black fixation cross and protruding 50 mm to the left and to the right of the screen midline were displayed in front of each participant. The distance between the left and the right placeholder was 320 mm. The squares were placed within a light-grey area, covering 200 mm^2^ of the screen. On each trial, one of them illuminated by turning white (74.6 cd/m^2^). Participants made their response by releasing the “home” button and touching the square that had illuminated. An RM Pentium PC custom software was used for the stimulus generation and recording of the responses.

### Design and Procedure

The experiment employed a 2 (movement congruency: congruent, incongruent)×2 (effector: hand, foot) fully within-participants design. A movement congruent action was one in which the participant reached out to the target that appears on the same side as the partner's previous response within an egocentric framework. For instance, reaching to the left when their co-actor had just reached to her left. Note that in keeping with some of the literature [24] we refer to the two levels of this factor as movement congruent/incongruent. However, when the confederate used her leg, actions were never congruent because the participant always responded with their hand. The dependent variable in both experiments was the time that elapsed between the target presentation and the target (i.e., screen) being touched.

All participants were tested individually and performed the task with the same confederate. The confederate always sat in the same position relative to the workspace (See Fig. 1). The experimenter verbally explained the instructions after which she performed a short demonstration of the procedure. The confederate's initial response triggered the target sequence in which co-actors alternated single responses. They were instructed to keep the home buttons pressed until a response was needed whilst at the same time fixating the cross in the centre of the screen. Then participants were required to reach out with their right hand and touch the target location, which illuminated for 100 ms. All trials had an inter-trial interval of 350 ms and a stimulus onset asynchrony (SOA) of approximately 1000 ms. Since SOA refers to the time between the release of the home button of Participant A and the target onset of Participant B, the duration of a trial varied slightly depending on individual differences in response speed. Participants performed two experimental blocks of 209 trials (i.e., 104 per participant plus the first trial which was not analysed since no response preceded it) by using their right hand to make the responses. However, in one of the blocks the confederate responded with her right hand whereas in the other – with her right foot (the block order was counterbalanced across participants). Regardless of which limb was used by the confederate, both the confederate and participant had a full view of each other and each other's targets and responses (See Fig. 1). Before commencing with the experiment, each pair completed a practice session of 21 trials. Participants were instructed to respond as quickly and as accurately as possible.

### Results and Discussion

**Figure 2:**
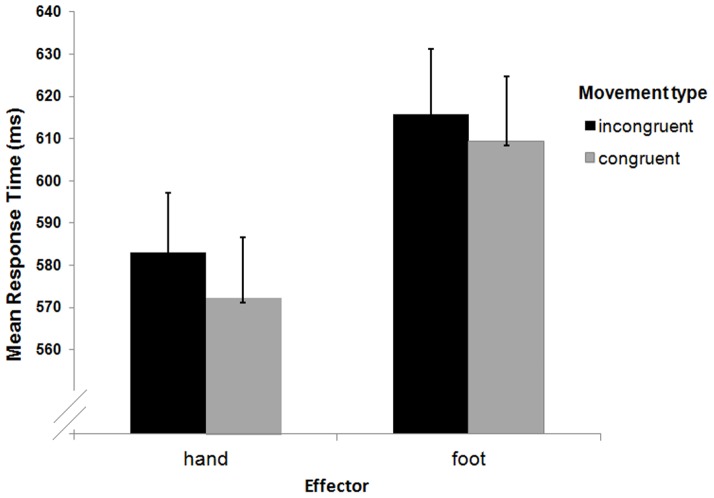
Mean RTs to localise targets as a function of effector and movement congruency in Experiment 1. Error bars represent standard errors of the mean.

RT outliers (more than two SDs above or below the mean) were removed prior to the formal analyses. Mean RTs were computed as a function of movement congruency (congruent, incongruent) and effector (hand, foot) and entered into a 2×2 fully-within participants ANOVA (See Fig. 2). The main effect of effector was significant (*F* (1, 20) = 30.92, p<.001, partial eta sq = .607). Thus, overall, participants were slower when the confederate responded with her foot as compared to the standard hand condition. The main effect of congruency was also significant (*F* (1, 20) = 17.75, p<.001, partial eta sq = .470). Finally, there was no reliable movement congruency *x* effector interaction (*F* (1, 20) = 3.89, *p*>.06, partial eta sq = .163). However, to test whether the movement congruency phenomenon was present in both conditions, we carried out follow-up comparisons. These analyses confirmed that participants exhibited a congruency effect in both the hand (*t* (1, 20) = 4.94, p<.001, Bonferroni adjusted alpha = .025) and the foot (*t* (1, 20) = 2.54, p<.02, Bonferroni adjusted alpha = .025) condition. No difference in within-participants' variability in RT across conditions was found (*F*s (1, 20)>0.57, *p*s>.307). Additionally, significant positive correlations emerged between the participants' and the confederate's responses in all four movement congruency-effector combinations (congruent, hands: *r* (19) = .53, *p*<.013; incongruent, hands: *r* (19) = .46, *p*<.035; congruent feet: *r* (19) = .54, *p*<.011; incongruent, feet: *r* (19) = .61, *p*<.003).

The results of Experiment 1 are consistent with previous work on the present joint-action effect; participants are generally faster to make a response that is congruent with their partner [22], [24], [27]. However, this effect occurred even when participants used a different effector to that used by their partner. This finding is not in line with the action co-representation account according to which action observation leads to automatic activation of motor representations in the observer [31], [24], [3], [4]. Thus, our results provide support for those reported by Dolk et al. [16] because emphasising the difference between the observed and the performed events should have prevented the integration of the partner's action into one's motor system.

Finally, the significant main effect of effector can be accounted for by the fact that the confederate was slower in the foot condition and this affected the participants' overall response tempo. Moreover, the significant relationships between participants' and confederate's responses reveal that participants, at some level, must have been taking into account their task-partner and their actions. Additionally, observing biological movements carried out by another individual has been reported to bias one's perception of timing [37], [38]. For example, Kaneko and Murakami [38] found that the speed of a stimulus was a significant predictor of how participants perceived observed motion so that the apparent duration proportionally increased with the speed logarithm.

## Experiment 2: Acting with attention-capturing cues, instead of a co-actor

Experiment 1 demonstrated that a common joint-action effect could occur even when the two task-partners engaged in very different actions. However, it could be argued that action co-representation was still occurring even when a different effector was used to that observed. For instance, the observed action could have been coded as “reaching to the right of their visual space”. Furthermore, even if participants did not represent the partner's actions per se, they may have coded the actor's action *intention* or *goal* [39]. Indeed, evidence exists showing that the movement congruency effect employed here may represent the intended goal [24] but see [22]. This is further supported by work suggesting that the MNS codes for intentions rather than body movement per se [40]. As Rizzolatti and colleagues [41], p. 25 argue, “For most mirror neurons, however, the relationship between the effective observed and executed motor acts is based on their common goal (e.g., grasping), regardless of how this goal is achieved”.

**Figure 3:**
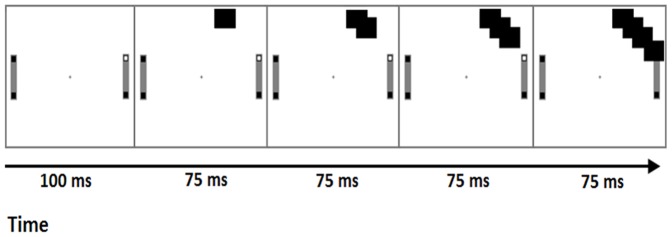
Trial sequence in Experiment 2.

In Experiment 2 therefore we examined whether the present movement congruency effect could be induced when participants performed the basic task but with no co-actor present, as in Dolk et al. [17]. Thus, where the partner would normally respond, attention- capturing cues moved across the display to the target (see Fig. 3).

### Methods

#### Participants

A volunteer sample of 20 (7 male; 13 female) participants aged between 19 and 32 (*M* = 22.50 years, *SD* = 3.17 years) took part in the study. All were undergraduates at the University of Essex, were right-handed, and naïve to the purposes of the study. They received £4 for their participation.

### Stimuli and Apparatus

The apparatus was as reported in Experiment 1. The black rectangular transients in the partner-absent condition had an area of 270 mm^2^. They were either displayed 40 mm to the left or 40 mm to the right of the screen midline, depending on which target location had illuminated on the partner's side of the table.

### Design and Procedure

The experiment employed a 2×2 fully within-participants design. One factor manipulated presence of co-actor (present, absent) whilst the other factor manipulated movement congruency (congruent, incongruent). “Congruency of action” in the co-actor-absent condition refers to, for instance, a rightward reaching response when the attention-capturing cues have also just moved to the right, as seen from the viewpoint of a co-actor had they been present.

The procedure in the co-actor-present condition was identical to the hand condition in Experiment 1 with the difference that two participants were tested simultaneously (i.e., there was no confederate in this experiment). In the co-actor-absent condition, however, only one of the participants was tested at a time, while the other was waiting with the experimenter. The participant's initial response triggered the target sequence in which the participant reached out and touched the target location, as in the co-actor-present condition. However, rather than a co-actor responding, a sequence of 4 black rectangular transients appeared (See Fig. 3). The first transient was displayed 100 ms after one of the target locations had illuminated. Every new transient appeared for 75 ms and then once the fourth transient reached and covered the target location, they began disappearing at 75 ms-intervals following a backward sequence. Each participant took part in two experimental blocks, i.e., the co-actor-present and absent conditions (209 trials in a block, 104 per person plus the first trial which was not analysed). The presentation order of the two blocks was counterbalanced. As in Experiment 1, participants always had a full view of their partner/rectangular transients, their targets and their responses. Participants first watched a demonstration by the experimenter and completed a 21-trial practice session. They were instructed to respond as quickly and as accurately as possible.

### Results and Discussion

**Figure 4:**
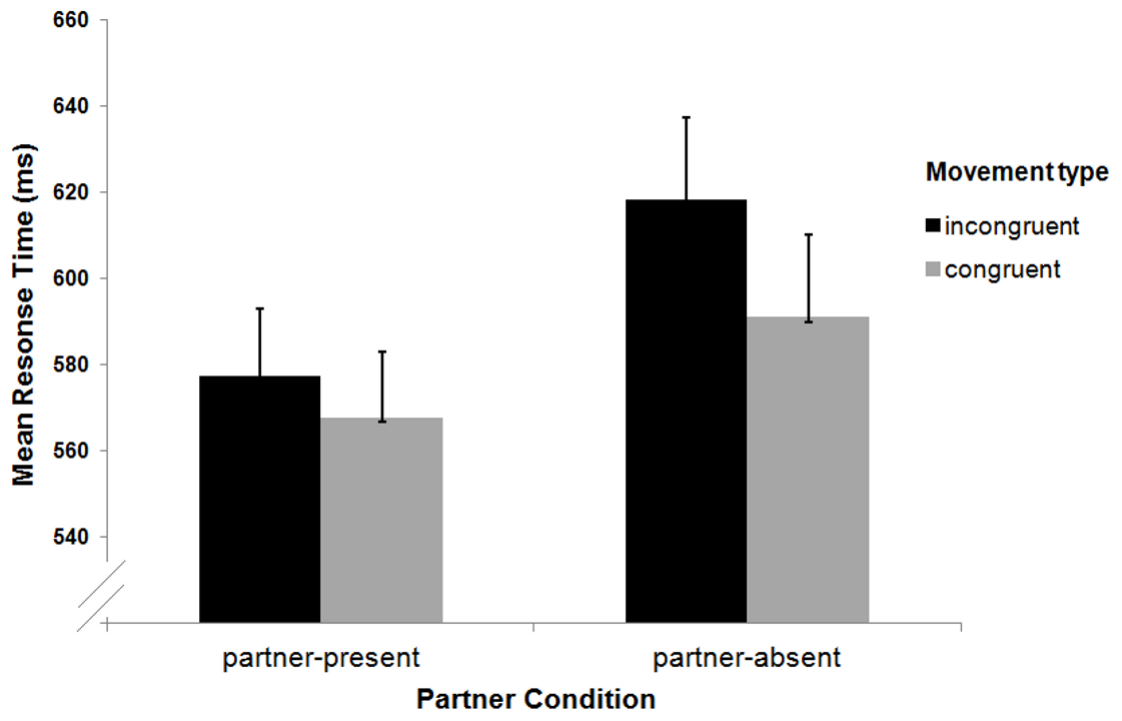
Mean RTs to localise targets as a function of partner and movement congruency in Experiment 2. Error bars represent standard errors of the mean.

As in Experiment 1 RT outliers (more than two SDs above or below the mean) were removed prior to the analyses. Mean RTs were computed as a function of movement congruency (congruent, incongruent) and partner (present, absent) and entered into a 2×2 fully-within participants ANOVA (See Fig. 4). The main effect of co-actor was significant (*F* (1, 19) = 5.65, p<.03, partial eta sq = .229). Thus, RTs were shorter when the participant performed with a co-actor than when they were responding alone. The main effect of movement congruency was also significant (*F* (1, 19) = 36.44, p<.001, partial eta sq = .657). Finally, there was a significant movement congruency *x* partner interaction (*F* (1, 19) = 7.89, p<.01, partial eta sq = .293). Planned follow-up comparisons revealed that the joint-action effect was present in both the co-actor-present (*t* (19) = 4.66, *p*<.001, Bonferroni adjusted alpha = .025) and the co-actor-absent conditions (*t* (19) = 4.67, *p*<.001, Bonferroni adjusted alpha = .025). Thus, essentially the interaction was driven by the significant difference in making a movement incongruent response when alone and when with a partner (*t* (19) = 3.00, *p*<.007, Bonferroni adjusted alpha = .025; See Fig. 4). There was no such difference between the two co-actor conditions when executing a congruent response (*p*>.115). Additionally, as in Experiment 1, we also examined whether there was a difference in within-participants' variability in RT as a function of condition. Again, none of the effects reached significance (*F*s (1, 19)>0.03, *p*s>.722). Moreover, although there was a significant movement congruency by co-actor interaction (*F* (1, 19) = 10.29, p<.005, partial eta sq = .351), none of the simple main effects was significant.

The results of Experiment 2 are in line with those reported by Dolk and colleagues [16], [17] since they showed that the presence of a co-actor is not necessary for a joint-action effect to arise. Moreover, the RT difference between making a movement congruent and movement incongruent action was somewhat bigger in the co-actor-absent condition (See Fig. 4). However, due to a greater RT variability in this condition, the inferential analyses indicated that the effect was similar to the one in the co-actor-present condition.

Interestingly, there was a significant difference in RT between making movement incongruent actions across the two partner conditions but no such difference occurred when initiating movement congruent actions. The latter fits well with the selective attention account according to which people are slower to respond to a previously attended location because they experience inhibition of return (IOR) [42], [43]. Furthermore, it could be assumed that the luminance transients were more attention-capturing than the arm/hand movements since they were high contrast and appeared abruptly. Indeed, abrupt visual onsets have long been known to effectively attract attention [44]–[47]. In sum, Experiment 2 provides evidence that a movement congruency joint-action effect can be driven by exogenous cues in the absence of a partner.

## General Discussion

Recent work has demonstrated that a well-established joint-action effect may not in fact be due to action co-representation. Dolk et al. [16], [17] showed that the Social Simon effect, can be generated when no partner is present. In the current experiments we examined whether another joint-action phenomenon, i.e., a movement congruency effect, may similarly be explained by a non-co-representation account. We have found that the effect emerged in two experiments even when action mirroring was not possible due to a difference between the observed and the performed action (Experiment 1) and when there was no partner present (Experiment 2). These results clearly do not support an explanation of the present movement congruency effect, based on action co-representation, since if automatic integration of the partner's actions was indeed driving the effect, it should have been abolished in the foot and co-actor-absent conditions.

The present findings can be placed within the context of other work challenging the notion that action co-representation, via the observer's motor system, drives joint-action effects. For instance, Vlainic et al. [15] showed that neither visual nor auditory information about the partner's actions was required for the Social Simon effect to occur. Furthermore, according to the Coordination Dynamics Approach, the vital component for such effects to occur is the emergent interpersonal motor coordination rather than the mental simulation of the observed action [48]–[51]. Moreover, when considering the interference in movement congruency paradigms, the proponents of this account suggest that rather than being indicative of “error” the motor system represents the necessary compensatory changes to ensure coordination across unequal kinematic requirements [50]. In support, Fine et al. [49] manipulated the spatial congruence between the participant and the confederate (i.e., whether they made horizontal or vertical movements) and the anatomical congruence (i.e., whether they were facing one another or the confederate was rotated at 90°). The results showed that *anatomical incongruence* did not create interference, suggesting that coordinating actions with the actor did not depend on the simulation of postural-based motor representations.

Rather than action co-representation, the present results seem to fit better with a more bottom-up explanation of this particular movement effect. One such explanation is IOR [43]. According to this account, a partner's reaching action shifts the observer's attention to the location of the response [22], [25]–[27]. Then, when the partner returns their hand, the observer's attention is shifted back to the centre of the display. Consequently, when a target appears at the responded-to location, participants inhibit the stimulus and/or a response to that position. Indeed, another way of describing the movement congruency effect is to say that RTs are *longer* when a participant is required to move to the same location where their partner just reached to. This “social IOR” account (or “between-person IOR”; [27]–[30]) predicts that any transient event that shifts an observer's attention will generate inhibition at that location, including for instance, a moving foot or moving transients as in our experiments. In support, human features, in general, easily attract attention. For example, hands have been found to affect the attentional prioritization of space [52]. Furthermore, we can assume that the foot also captured participants' attention because of it being more unusual than a hand. Along the same lines, the visual transients in Experiment 2 are likely to have automatically attracted the observer's attention because of their abrupt motion [46], [47]. Thus, although moving transients replaced the biological partner in Experiment 2, what may be important is the introduction of an event that is salient enough to produce an attentional shift to that location. This explains why the effect occurs even when the partner's targets and final part of the response (i.e., arm reach) are occluded from view – the actor's hand movement and gaze shift are enough to direct the observer's attention to that direction [25], [28].

We can only speculate about the neural basis of social IOR since to the best of our knowledge there have not been any published neuropsychological data on it. Still, behavioral research has identified many similarities between basic IOR and its social counterpart. For example, in a series of experiments Skarratt and colleagues [25] demonstrated that, similarly to IOR, social IOR arises during the perceptuo-attentional and/or motor programming stages, prior to response initiation. Additionally, Welsh et al. [29] reported a significant correlation between these two effects. ERP studies have indicated that IOR is associated with a modulation of early perceptual processing since a significant amplitude reduction in the P1 and/or N1 signals is usually observed during IOR tasks [53]–[57]. Additionally, results from neuroimaging studies have revealed that the potential neural correlates of spatial IOR could be found in a dorsal frontoparietal network in the brain which includes the frontal eye field and the superior parietal cortex [58]–[61]. Thus, if social IOR is indeed an IOR effect, it should comprise an attentional and an oculomotor component.

An alternative bottom-up account is a variant of the referential coding theory that Dolk and colleagues [16], [17] utilised to explain the occurrence of a Social Simon effect. According to the referential coding account, when a sufficiently salient event affords the referential coding of the response, participants code their responses in relation to that event [17]. This mode of representing spatial relations is *egocentric* since referential coding is formed from the observer's perspective (i.e., subject-to-object relation) [62]. Applying this to the present paradigm, instead of co-representing the co-actor's actions per se, a participant's attention may have been attracted to their co-actor's response position as a result of the response and target appearance. This initiated a code in which the responded-to location became a reference point. For instance, when the co-actor reached to the participant's left, this could have set up a code that facilitated the representation of right, giving rise to reduced RTs to right-hand targets which induced the basic congruency effect. Furthermore, as in the Social Simon effect, the horizontal dimension is a salient aspect in our paradigm [17] and, as suggested by Hommel et al. [5], the occurrence of another event along the same dimension should increase the salience of the task and provide a stronger referential landmark for coding.

## Conclusion

In sum, we have demonstrated that a common joint-action effect can occur even in the absence of a co-actor. While it has previously been proposed that in joint-action studies individuals are co-representing each other's actions, the present findings indicate that a particular movement congruency effect does not rely on the “socialness” of the co-actor. Indeed, referring to the present arm movement phenomenon as a “movement congruency effect” [24] appears to be a mislabel since it may be due to IOR rather than congruency of movement.

### Data Availability

The data for the current research is freely available upon request by contacting the corresponding author Silviya P. Doneva (sdoneva@essex.ac.uk).

## References

[1] Atmaca S, Sebanz N, Prinz W, Knoblich G (2008) Action co-representation: The joint SNARC effect. Social Neuroscience 3: 410–420. 18633833 10.1080/17470910801900908

[2] Liepelt R, Stenzel A, Lappe M (2012) Specifying social cognitive processes with a social dual-task paradigm. Frontiers in Human Neuroscience 6: 86. 22783178 10.3389/fnhum.2012.00086 PMC3390591

[3] Sebanz N, Knoblich G, Prinz W (2003) Representing others' actions: Just like one's own?. Cognition 88: B11–B21. 12804818 10.1016/s0010-0277(03)00043-x

[4] Sebanz N, Knoblich G, Prinz W (2005) How two share a task. Journal of Experimental Psychology: Human Perception and Performance 31: 1234–1246. 16366786 10.1037/0096-1523.31.6.1234

[5] Hommel B, Müsseler J, Aschersleben G, Prinz W (2001) The theory of event coding (TEC): a framework for perception and action planning. Journal of Behavioral and Brain Science 24: 849–878. 10.1017/s0140525x01000103 12239891

[6] Kornblum S, Hasbroucq T, Osman A (1990) Dimensional overlap: Cognitive basis for stimulus–response compatibility—a model and taxonomy. Psychological Review 97: 253–270. 2186425 10.1037/0033-295x.97.2.253

[7] Hommel B (1996) S-R compatibility effects without response uncertainty. Quarterly Journal of Experimental Psychology 49A: 546–571.

[8] Fogassi L, Ferrari P, Gesierich B, Rozzi S, Chersi F, et al (2005) Parietal Lobe: From Action Organization to Intention Understanding. Science 308 ((5722)) 662–667. 15860620 10.1126/science.1106138

[9] Rizzolatti G, Fadiga L, Gallese V, Fogassi L (1996) Premotor cortex and the recognition of motor actions. Cognitive Brain Research 3 ((2)) 131–142. 8713554 10.1016/0926-6410(95)00038-0

[10] Mukamel R, Ekstrom A, Kaplan J, Iacoboni M, Fried I (2010) Single-Neuron Responses in Humans during Execution and Observation of Actions. Current Biology 20 ((8)) 750–756. 20381353 10.1016/j.cub.2010.02.045 PMC2904852

[11] Hamilton A, Wolpert D, Frith U, Grafton S (2006) Where does your own action influence your perception of another person's action in the brain?. Neuroimage 29 ((2)) 524–535. 16112877 10.1016/j.neuroimage.2005.07.037

[12] Frischen A, Loach D, Tipper S (2009) Seeing the world through another person's eyes: simulating selective attention via action observation. Cognition 111 ((2)) 212–218. 19296931 10.1016/j.cognition.2009.02.003

[13] Hickok G (2009) Eight Problems for the Mirror Neuron Theory of Action Understanding in Monkeys and Humans. Journal Of Cognitive Neuroscience 21 ((7)) 1229–1243. 19199415 10.1162/jocn.2009.21189 PMC2773693

[14] Lingnau A, Gesierich B, Caramazza A (2009) Asymmetric fMRI adaptation reveals no evidence for mirror neurons in humans. Proceedings Of The National Academy Of Sciences Of The United States Of America 106 ((24)) 9925–9930. 19497880 10.1073/pnas.0902262106 PMC2701024

[15] Vlainic E, Liepelt R, Colzato LS, Prinz W, Hommel B (2010) The virtual co-actor: The social Simon effect does not rely on online feedback from the other. Frontiers in Psychology 1: 208. 21833264 10.3389/fpsyg.2010.00208 PMC3153814

[16] Dolk T, Hommel B, Colzato LS, Schütz–Bosbach S, Prinz W, et al (2011) How ‘social’ is the social Simon effect? Frontiers in Psychology 2: 84. 21687453 10.3389/fpsyg.2011.00084 PMC3110342

[17] Dolk T, Hommel B, Prinz W, Liepelt R (2013) The (Not So) Social Simon Effect: A Referential Coding Account. Journal of Experimental Psychology: Human Perception and Performance 39: 1248–1260. 23339346 10.1037/a0031031

[18] Botvinick M, Cohen J (1998) Rubber hands “feel” touch that eyes see. Nature 391: 756. 9486643 10.1038/35784

[19] Müller BN, Brass M, Kühn S, Tsai C, Nieuwboer W, et al (2011) When Pinocchio acts like a human, a wooden hand becomes embodied. Action co-representation for non-biological agents. Neuropsychologia 49 ((5)) 1373–1377. 21241722 10.1016/j.neuropsychologia.2011.01.022

[20] Tsai CC, Kuo WJ, Hung DL, Tzeng OJ (2008) Action co-representation is tuned to other humans. Journal of Cognitive Neuroscience 20: 2015–2024. 18416679 10.1162/jocn.2008.20144

[21] Hommel B (1993) The role of attention for the Simon effect. Psychological Research 55: 208–222. 8416040 10.1007/BF00419608

[22] Cole GG, Skarratt P, Billing R (2012) Do action goals mediate social inhibition of return?. Psychological Research 76 ((6)) 736–746. 22143901 10.1007/s00426-011-0395-7

[23] Hayes S, Hansen S, Elliott D (2010) Between nervous system effects on attention and action: Joe and Fred revisited. Psychological Research 74: 302–312. 19603181 10.1007/s00426-009-0250-2

[24] Ondobaka S, de Lange FP, Newman-Norlund RD, Wiemers M, Bekkering H (2012) Interplay Between Action and Movement Intentions During Social Interaction. Psychological Science (Sage Publications Inc.) 23 ((1)) 30–35. 10.1177/0956797611424163 22157675

[25] Skarratt PA, Cole GG, Kingstone A (2010) Social inhibition of return. Acta Psychologica 134 ((1)) 48–54. 20044064 10.1016/j.actpsy.2009.12.003

[26] Skarratt PA, Cole GG, Kuhn G (2012) Visual cognition during real social interaction. Frontiers in Human Neuroscience 6: 1–9. 22754521 10.3389/fnhum.2012.00196 PMC3386564

[27] Welsh TN, Elliot D, Anson JG, Dhillon V, Weeks DJ, et al (2005) Does Joe influence Freds actions? Inhibition of return across different nervous systems. Neuroscience Letters 385: 99–104. 15927370 10.1016/j.neulet.2005.05.013

[28] Welsh TN, Lyons J, Weeks DJ, Anson JG, Chua R, et al (2007) Within- and between-person inhibition of return: Observation is as good as performance. Psychonomic Bulletin and Review 14: 950–956. 18087965 10.3758/bf03194127

[29] Welsh TN, McDougall LM, Weeks DJ (2009a) The performance and observation of action shape future behaviour. Brain and Cognition 71: 64–71. 19406547 10.1016/j.bandc.2009.04.001

[30] Welsh TN, Ray MC, Weeks DJ, Dewey D, Elliott D (2009b) Does Joe influence Fred's action? Not if Fred has autism spectrum disorder. Brain Research 1248: 141–148. 19028469 10.1016/j.brainres.2008.10.077

[31] Brass M, Bekkering H, Prinz W (2001) Movement observation affects movement execution in a simple response task. Acta Psychologica 106 ((1–2)) 3–22. 11256338 10.1016/s0001-6918(00)00024-x

[32] Liepelt R, von Cramon DY, Brass M (2008) What is matched in direct matching? Intention attribution modulates motor priming. Journal of Experimental Psychology: Human Perception and Performance 34: 578–591. 18505325 10.1037/0096-1523.34.3.578

[33] Sebanz N, Bekkering H, Knoblich G (2006) Joint action: bodies and minds moving together. Trends In Cognitive Sciences 10 ((2)) 70–76. 16406326 10.1016/j.tics.2005.12.009

[34] Rizzolatti G, Craighero L (2004) The mirror-neuron system. Annual Reviews of Neuroscience 27: 169–192. 10.1146/annurev.neuro.27.070203.144230 15217330

[35] Avenanti A, Sirigu A, Aglioti S (2010) Racial bias reduces empathic sensorimotor resonance with other-race pain. Current Biology: CB 20 ((11)) 1018–1022. 20537539 10.1016/j.cub.2010.03.071

[36] Hommel B, Colzato LS, van den Wildenberg WPM (2009) How social are task representations? Psychological Science 20: 794–798. 19493327 10.1111/j.1467-9280.2009.02367.x

[37] Watanabe K (2008) Behavioral speed contagion: Automatic modulation of movement timing by observation of body movements. Cognition 106 ((3)) 1514–1524. 17612518 10.1016/j.cognition.2007.06.001

[38] Kaneko S, Murakami I (2009) Perceived duration of visual motion increases with speed. Journal Of Vision 9 ((7):14) 1–12. 10.1167/9.7.14 19761329

[39] Wilson M, Knoblich G (2005) The case for motor involvement in perceiving conspecifics. Psychological Bulletin 131: 460–473. 15869341 10.1037/0033-2909.131.3.460

[40] Kohler E, Keysers C, Umilta MA, Fogassi L, Gallese V, et al (2002) Hearing sounds, understanding actions: Action representation in mirror neurons. Science 297: 846–848. 12161656 10.1126/science.1070311

[41] Rizzolatti G, Fabbri-Destro M, Cattaneo L (2009) Mirror neurons and their clinical relevance. Nature Clinical Practice. Neurology 5 ((1)) 24–34. 19129788 10.1038/ncpneuro0990

[42] Kingstone A, Pratt J (1999) Inhibition of return is composed of attentional and oculomotor processes. Perception and Psychophysics 61: 1046–1054. 10497426 10.3758/bf03207612

[43] Posner MI, Cohen Y (1984) Components of visual orienting. In: Bouma, H., Bouwhuis, D. (Eds.) Attention and Performance, vol. X. Lawrence Erlbaum, London: 531–554.

[44] Cole GG, Kuhn G (2009) Appearance matters: attentional orienting by new objects in the precuing paradigm. Visual Cognition 17: 755–776.

[45] Cole GG, Kuhn G (2010) Attentional capture by object appearance and disappearance. Quarterly Journal of Experimental Psychology 63: 147–159. 10.1080/17470210902853522 19396733

[46] Ruz M, Lupianez J (2002) A review of attentional capture: On it's automaticity and sensitivity to endogenous control. Psicologica 23: 283–309.

[47] Yantis S, Jonides J (1984) Abrupt visual onsets and selective attention: Evidence from visual search. Journal of Experimental Psychology: Human Perception & Performance 10: 601–621. 6238122 10.1037//0096-1523.10.5.601

[48] Fine JM, Amazeen EL (2011) Interpersonal Fitts's law: When two perform as one. Experimental Brain Research 211: 459–469. 21547558 10.1007/s00221-011-2707-y

[49] Fine JM, Gibbons CT, Amazeen EL (2013) Congruency effects in interpersonal coordination. Journal Of Experimental Psychology: Human Perception And Performance 39 ((6)) 1541–1556. 23458094 10.1037/a0031953

[50] Richardson MJ, Campbell W, Schmidt RC (2009) Movement interference during action observation as emergent coordination. Neuroscience Letters 449: 117–122. 18996439 10.1016/j.neulet.2008.10.092

[51] Romero V, Coey C, Schmidt RC, Richardson MJ (2012) Movement Coordination or Movement Interference: Visual Tracking and Spontaneous Coordination Modulate Rhythmic Movement Interference. PLoS ONE 7 ((9)) e44761. 23028607 10.1371/journal.pone.0044761 PMC3444463

[52] Reed CL, Grubb JD, Steele C (2006) Hands up: Attentional prioritization of space near the hand. Journal Of Experimental Psychology: Human Perception And Performance 32 ((1)) 166–177. 16478334 10.1037/0096-1523.32.1.166

[53] Hopfinger J, Mangun G (2001) Tracking the influence of reflexive attention on sensory and cognitive processing. Cognitive, Affective & Behavioral Neuroscience 1 ((1)) 56–65. 10.3758/cabn.1.1.56 12467103

[54] Prime D, Ward L (2004) Inhibition of return from stimulus to response. Psychological Science 15 ((4)) 272–276. 15043647 10.1111/j.0956-7976.2004.00665.x

[55] Prime D, Ward L (2006) Cortical expressions of inhibition of return. Brain Research 1072 ((1)) 161–174. 16445889 10.1016/j.brainres.2005.11.081

[56] Van der Lubbe R, Vogel R, Postma A (2005) Different effects of exogenous cues in a visual detection and discrimination task: delayed attention withdrawal and/or speeded motor inhibition?. Journal Of Cognitive Neuroscience 17 ((12)) 1829–1840. 16356322 10.1162/089892905775008634

[57] Wascher E, Tipper SP (2004) Revealing effects of noninformative spatial cues: An EEG study of inhibition of return. Psychophysiology 41 ((5)) 716–728. 15318878 10.1111/j.1469-8986.2004.00198.x

[58] Lepsien J, Pollmann S (2002) Covert reorienting and inhibition of return: An event-related fMRI study. Journal of Cognitive Neuroscience 14: 127–144. 11970781 10.1162/089892902317236795

[59] Mayer AR, Dorflinger JM, Rao SM, Seidenberg M (2004) Neural networks underlying endogenous and exogenous visual-spatial orienting. Neuroimage 23: 534–541. 15488402 10.1016/j.neuroimage.2004.06.027

[60] Mayer AR, Seidenberg M, Dorflinger JM, Rao SM (2004) An event-related fMRI study of exogenous orienting: Supporting evidence for the cortical basis of inhibition of return? Journal of Cognitive Neuroscience 16: 1262–1271. 15453978 10.1162/0898929041920531

[61] Müller NG, Kleinschmidt A (2007) Temporal dynamics of the attentional spotlight: Neuronal correlates of attentional capture and inhibition of return in early visual cortex. Journal of Cognitive Neuroscience 19: 587–593. 17381250 10.1162/jocn.2007.19.4.587

[62] Zaehle T, Jordan K, Wüstenberg T, Baudewig J, Dechent P, et al (2007) The neural basis of the egocentric and allocentric spatial frame of reference. Brain Research 1137 ((4)) 92–103. 17258693 10.1016/j.brainres.2006.12.044

